# Supplementary material for: Precise tumor immune rewiring via synthetic CRISPRa circuits gated by concurrent gain/loss of transcription factors
Source: Nat Commun. 2022 Mar 18;13:1454. doi: 10.1038/s41467-022-29120-y (PMC8933567; doi:10.1038/s41467-022-29120-y)
Supplement: Supplementary file 1 — Supplementary Information [file 41467_2022_29120_MOESM1_ESM.pdf]

SUPPLEMENTARY INFORMATION

Supplementary Fig. 1: An active CRISPRa can flexibly program immunostimulatory outputs in tumor cells.

Supplementary Figure 2: Construction of a preliminary dual-input, AND-NOT logic circuit.

Supplementary Figure 3: Construction of a customized AND-NOT logic circuit (v1) targeting a malignant state.

Supplementary Figure 4: Development of a more accurate NOT gate for CRISPRa by employing the anti-CRISPR AcrIIA4.

Supplementary Figure 5: An improved P_{Suv}/P_{M2} AND-NOT logic circuit (v2) rewires p53-deficient tumor cells to produce immunostimulatory ligands.

Supplementary Figure 6: The P_{Suv}/P_{M2} AND-NOT circuit (v2) empowers immune rewiring of p53-deficient tumors.

Supplementary Table 1: Reagents or resources.

Supplementary Table 2: Spacer sequence of gRNAs.

Supplementary Table 3: Genotyping primers.

Supplementary Table 4: qPCR primers.

Supplementary Data 1: Sequences from key constructs. – This file is provided in a separate zipped word file.

Supplementary Figures and Legends:

Supplementary Fig. 1

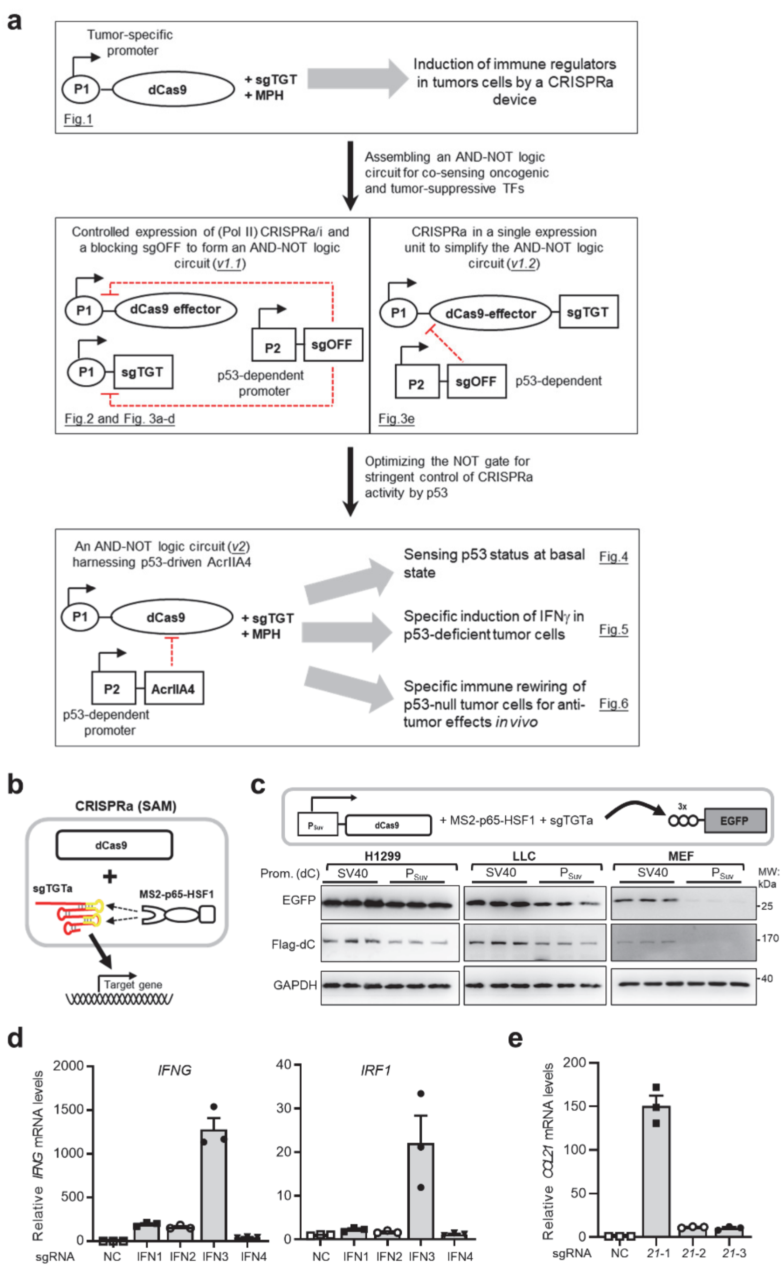

Supplementary Figure 1: An active CRISPRa can flexibly program immunostimulatory outputs in tumor cells. **(a)** A schematic for the overall flow of the experiments. It shows the stepwise development for a new class of highly specific tumor-rewiring gene circuit. **(b)** The illustration shows the components for the CRISPRa SAM effector used in the

present study. **(c)** Tumor cells (H1299, LLC) or normal mouse embryonic fibroblasts (MEFs) were transfected with different sets of CRISPRa plasmids featuring dCas9 either under a constitutive SV40 promoter or a tumor-specific promoter (of surviving gene, P_{Suv}). The targeting sgRNA (sgTGTa) was designed for a reporter construct with 3x matched sequences upstream of a minimal promoter-led EGFP. The amounts of plasmids in the transfection mix remained constant for different cell types. The levels of Flag-dCas9 ("dC") and EGFP were determined by IB. Replicated samples were from individual transfections. The results are representative of 2 independent experiments. **(d)** H1299 cells in each well were transfected with P_{Suv}-dCas9, MPH and a different sgRNA targeting sequences upstream of *IFNG* ("IFN1" to "IFN4"). 30 h after transfection, the cells were harvested for qPCR analyses. **(e)** H1299 cells were transfected with the CRISPRa plasmid mix containing different sgRNAs targeting sequences upstream of *CCL21* (denoted as "*21*-1", "*21*-2" and "*21*-3"). 24 h after transfection, the cells were harvested for qPCR analyses. In this figure the qPCR results are presented as mean±SEM (n=3, biological replicates). Source data are provided in the Source Data file.

Supplementary Fig. 2

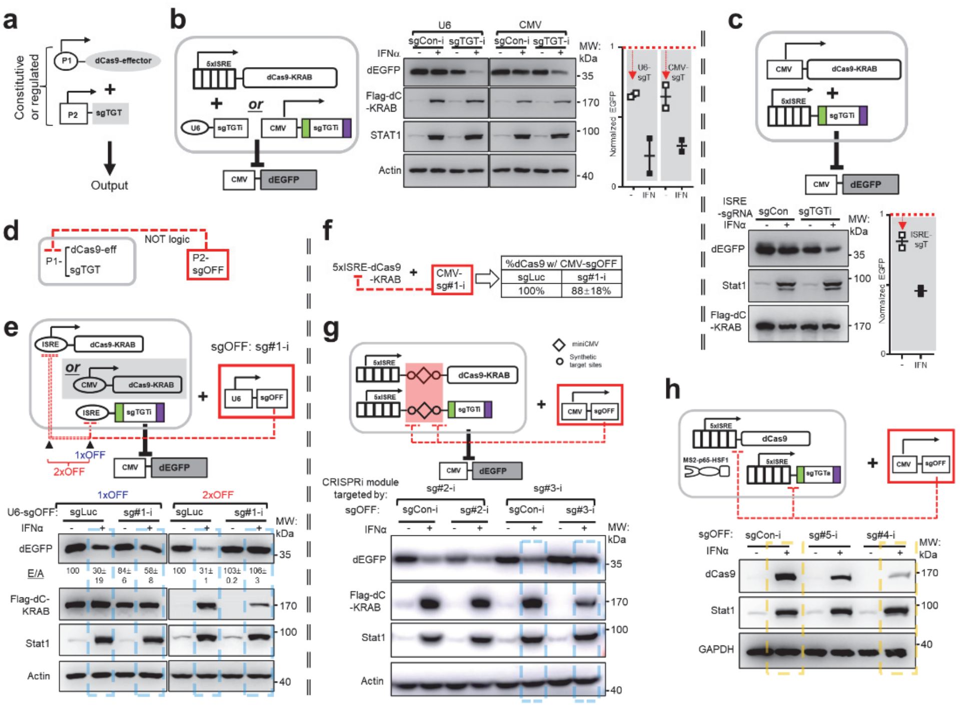

Supplementary Figure 2: Construction of a preliminary dual-input, AND-NOT logic circuit. **(a)** The illustration shows the design of using two promoters (P1, P2) to respectively drive dCas9 and sgRNA, forming the basis for an AND logic circuit (sensing two inputs), or for flexibly regulating CRISPRa/i activities (via regulation of either the dCas9 part or sgTGT). **(b)** The left illustration shows Pol II promoter (CMV) -driven targeting sgRNA for inhibition (sgTGTi) for suppressing transcription of an CMV-driven destabilized EGFP (dEGFP) reporter via a unique sequence near its minimal promoter. The U6-sgTGTi was used as a positive control. The dCas9-KRAB (Flag-tagged) is led by 5x ISRE to respond to IFN treatment. Constructs of non-targeting sgRNA (sgCon-i) were used as negative controls. Circuit-transfected 293T cells were treated with indicated doses of IFNα for 24 h and were harvested for IB analyses (middle). The ratios of dEGFP/Actin were quantitated. The levels in the

sgTGTi groups (±IFN) are presented relative to the levels in the untreated sgCon-i group (right, red arrows indicating basal CRISPRi activity without IFN signal). **(c)** A circuit parallel to those described above was constructed with a constitutive dCas9-KRAB (CMV) and a regulated sgTGTi (5xISRE) targeting dEGFP. The ratios of dEGFP/Flag were quantitated. The quantitation is presented similarly as in (b). **(d)** The illustration shows a strategy of using a P2-driven off-switching sgRNA (sgOFF) to form a NOT gate against a CRISPRa/i (eff: effector). **(e)** The top illustration shows a circuit consisting of a CRISPRi with 5xISRE-co-regulated components, in conjunction with another U6-sgOFF. In this case, a dual-target repression module ("2xOFF") is formed. An alternative circuit is also designed to feature CMV-dCas9-KRAB (highlighted in grey) and an ISRE-sgTGTi, with the latter as the only target for the U6-sgOFF ("1xOFF"). Here, a specific sgOFF ("sg#1-i") targeting a sequence derived from the ISRE promoter was used. The 293T cells were transfected with the circuit (a non-targeting sgLuc as a control for sg#1-i). The cells were treated with ± 500 IU/ml IFN for 48 h before harvested for IB (lower panel). The relative ratios of EGFP/Actin are marked in the panel. **(f)** CMV-sg#1-i was transfected together with 5xISRE-dCas9-KRAB to test its activity in suppressing the expression of dCas9-KRAB. The transfections and treatments were carried out similarly as above. The levels of dCas9-KRAB levels in IFN-treated, sg#1-i-transfected cells were presented relative to the control sgRNA group (%, n=2, ±range). **(g)** The illustration on the left shows a circuit where the CRISPRi actuator is driven by 5x ISRE promoter and the sgOFF is driven by a CMV promoter. To optimize the inhibitory effect by sgOFF, we engineered multiple

target sites near the transcriptional start of dCas9-KRAB and sgTGTi. For comparison, two different target sequences (respectively corresponding to sg#2-i and sg#3-i) were tested. Cells and samples were handled similarly as in (e). **(h)** An inducible CRISPRa SAM complex (with dCas9 and sgTGTa led by 5x ISRE, and constitutive expression of MPH) is also subjected to inhibition by a CMV-driven sgOFF (illustration in the upper panel). Here, two sgOFF (sg#4-i and sg#5-i) were designed against unique sequences in the 5xISRE-dCas9 and -sgRNA promoters, corresponding to the junctions of miniCMV/multiple cloning site and of ISRE/miniCMV, respectively. Transfected cells were treated with ±1000 IU/ml of IFN for 36 h. The cell lysates were examined by IB, for the effects by the two sgOFFs to limit dCas9 induction (lower panel). The blotting results in (b), (c), (e), (g) and (h) are representative of 2 independent experiments. In quantitation shown in (b), (c), (e) and (f), the values of mean±range are presented (n=2 independent experiments). Source data are provided in the Source Data file.

Supplementary Fig. 3

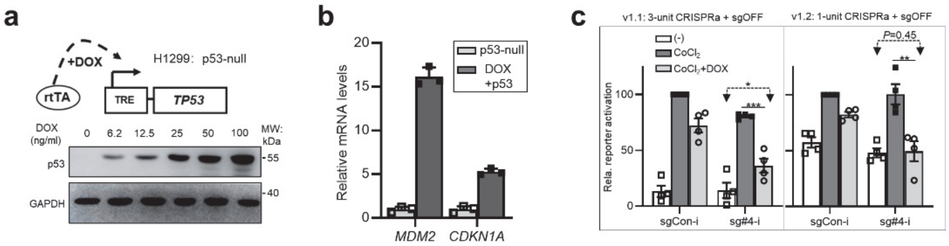

Supplementary Figure 3: Construction of a customized AND-NOT logic circuit (v1) targeting a malignant state. **(a)** The p53-null H1299 cells were introduced with tetracycline-inducible p53 via a lentiviral vector. Cells were treated with indicated doses of DOX for 48 h and the cell lysates were analyzed on IB (a representative of 2 independent experiments). **(b)** These cells were treated with DOX (50 ng/ml) for 24 h and the RNA samples were analyzed by qPCR (mean±SEM, n=3 biological replicates). **(c)** The performances by the AND-NOT circuits either based on the 3-unit or 1-unit CRISPRa (v1.1 or v1.2) was determined in p53-tet cells [related to Fig. 3d, e] (mean±SEM, n=4 measurements from independent experiments). One-sided t-tests were performed (*: $P<0.05$ [0.027]; **: $P<0.01$ [0.0035]; ***: $P<0.001$ [0.00023]). Source data are provided in the Source Data file.

Supplementary Fig. 4

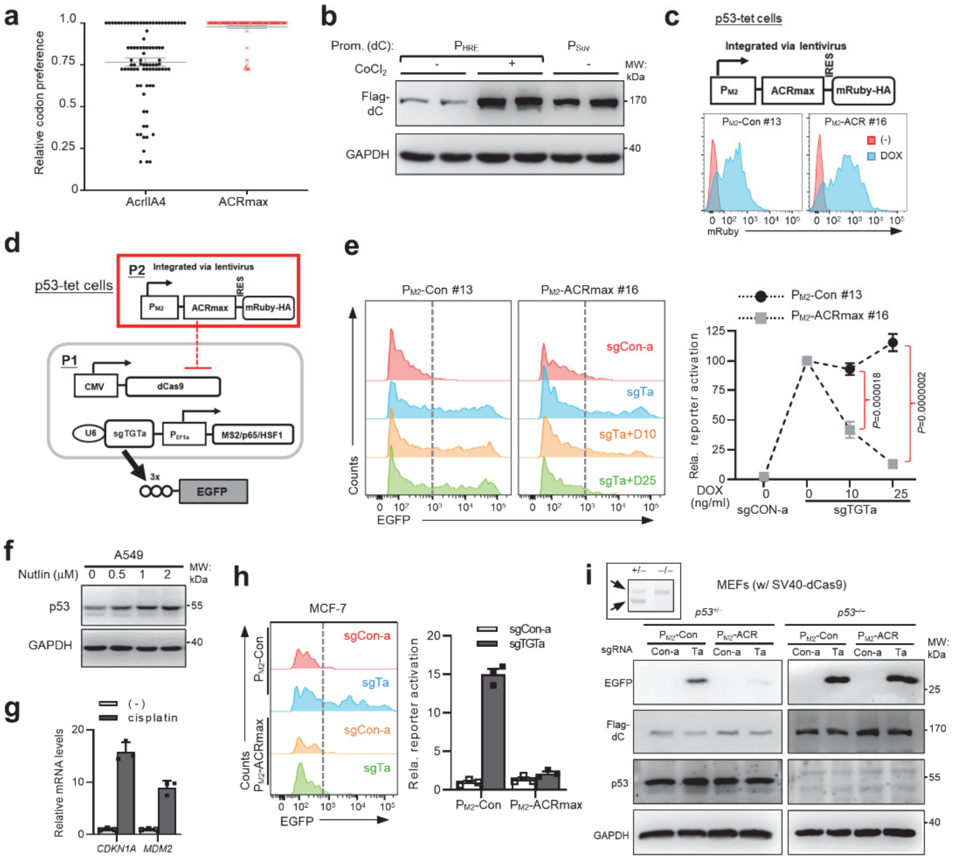

Supplementary Figure 4: Development of a more accurate NOT gate for CRISPRa by employing the anti-CRISPR AcrIIA4. **(a)** Codon usage for *AcrIIA4* is shown in the top graph. Each codon in the original *AcrIIA4* (*L. monocytogenes*) was compared to the most preferred ones in human and their fractional ratios (≤1) were presented. The marked increases of such ratios can be seen following human-codon optimization (ACRmax). **(b)** To analyze the activities by onco-TF-associated promoters, p53-tet cells were transfected with dCas9 constructs either led by 3x HRE or by P_{Suv}. Some HRE-dCas9-transfected cells were treated with 150 μM $CoCl_2$ for 24 h. The cell lysates were analyzed by IB. Replicated samples in the results were from 2 parallel transfections. **(c-e)** In (c), the top illustration shows that the p53-tet H1299 cells were introduced with

the P_{M2}-ACRmax-mRuby inhibitory module (or the non-expression control) via lentivirus. Two good DOX responder clones (mRuby induction) were picked for further analyses (bottom). (d and e) Cells from these clones were further transfected with constructs for a strong CRISPRa complex (sgCon-a as a control), together with the EGFP reporter (illustrated in (d)). (e) Cells were treated with indicated doses of DOX for 24 h and were harvested for flow cytometry analysis for EGFP fluorescence. On the left, the histogram shows the fluorescence pattern for $EGFP^+$ cells ("sgTa" and "D" denoting sgTGTa and DOX, respectively). The dotted lines mark high levels of EGFP positivity definitively attributed to CRISPRa activity. The quantitation is shown on the right ($EGFP^+$%×MFI, mean±SD, n=4 biological replicates). Two-sided Student's t-tests were performed for indicated groups (*P* values provided). **(f and g)** A549 cells were treated with indicated doses of Nutlin (f) or cisplatin (g). The cells were harvested for IB and qPCR analyses, respectively. Results in (f) are representative of 2 independent experiments. In (g), the qPCR results as mean±SD (n=3 biological replicates). **(h)** A circuit with a strong CRISPRa actuator (CMV-dCas9 and U6-sgTGTa (sgTa)) and an inhibitory module of P_{M2}-ACRmax was introduced into MCF-7 cells. Similar to results in (e), a representative histogram shows the fluorescence pattern for $EGFP^+$ cells (left). The quantitation of MFI is shown next to the histogram (mean±SEM, n=3 biological replicates). **(i)** The $p53^{+/-}$ or $p53^{-/-}$ MEFs were prepared. The inset shows a representative genotype analysis. The circuit transfected was similar to the one used in (h), except that another constitutive dCas9 construct (under SV40 promoter) was adopted. The cells lysates were subjected to IB. The results are representative of 2

independent experiments. Source data are provided in the Source Data file.

Supplementary Fig. 5

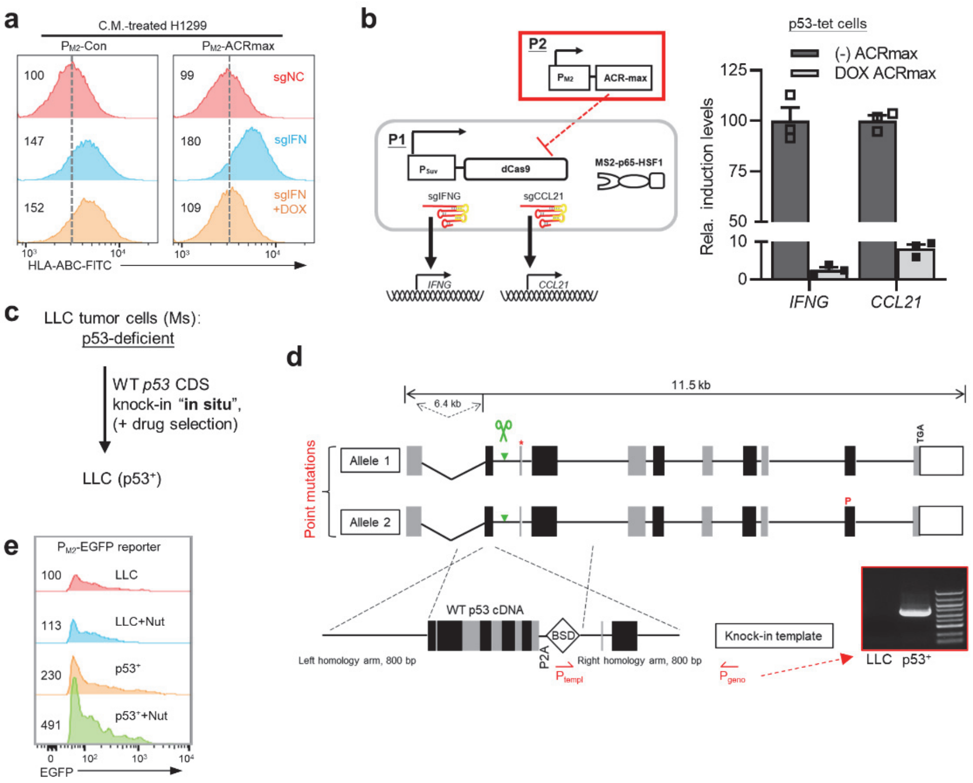

Supplementary Figure 5: An improved PSuv/PM2 AND-NOT logic circuit (v2) rewires p53-deficient tumor cells to produce immunostimulatory ligands. **(a)** The p53-tet H1299 cells (± 10 ng/ml DOX) were introduced with the circuit shown in Fig. 5a. The PSuv-driven CRISPRa is programmed to activate transcription of endogenous *IFNG*, whereas PM2-ACRmax forms an inhibitory module (compared to a non-expressor construct, PM2-Con). The non-targeting sgNC was used as a negative control for circuit actuation. The conditioned media from the transfected cells were collected and were added to unmodified H1299 cells. The levels of Class I HLA were determined. The relative MFI values are marked on the histograms. The dotted lines denote the control levels. **(b)** The p53-tet cells were introduced with a circuit similar that involved in (a), except that the circuit is programmed (with sgIFN3 and sgCCL21-1) to activate

transcription of endogenous *IFNG* and *CCL21* in a multiplexed manner (left illustration). The cells were harvested 24 h after transfection and were subjected to qPCR analyses (right). The qPCR results are presented as mean±SEM (n=3 biological replicates). **(c-e)** The p53-deficient mouse Lewis lung carcinoma cells and their p53-rescued derivatives were used additionally to test the performance of the AND-NOT circuit. The strategy for preparing the p53-rescued LLC cells is provided in (c). (d) The illustration shows the *p53* gene structure, along with the WT *p53* knock-in template (with 5' and 3' homology arms). cDNA sequencing confirmed a non-sense (E32, red asterisk) and a R334P (red "P") mutations on different alleles of *p53* in LLC cells. Green arrowheads show the Cas9/sgRNA target site in *p53* gene. The selective marker for Blasticidin (BSD) was engineering in the repair template. A genotyping strategy is shown, with a forward primer (P_{templ}) for part of the repair template sequence and a reverse primer (P_{geno}) complementary to the genomic sequence to the 3' of the insert. Only the samples from the $p53^+$, but not the parental cells, showed positive amplification. (e) The parental and the $p53^+$ LLC cells were transfected with P_{M2}-EGFP reporter. Cells were treated ± Nutlin and subjected to flow cytometry. The fluorescence of $EGFP^+$ cells are shown. Relative $EGFP^+$%×MFI levels are presented on the histogram. Source data are provided in the Source Data file.

Supplementary Fig. 6

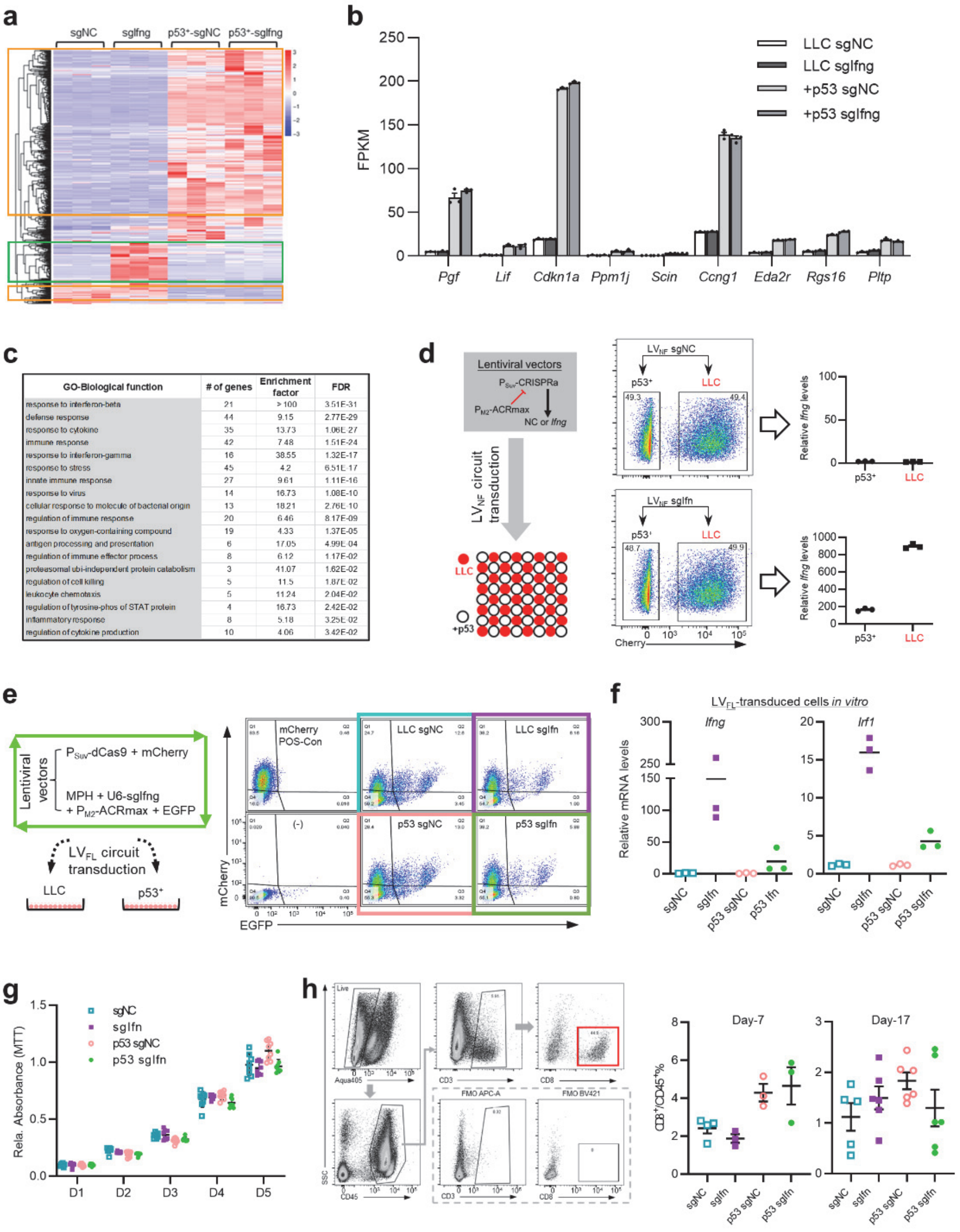

Supplementary Figure 6: The PSuv/PM2 AND-NOT circuit (v2) empowers immune rewiring of p53-deficient tumors. **(a-c)** The parental and p53$^+$ LLC cells were transfected with the PSuv/PM2 circuit (v2) for conditional *Ifng* activation by CRISPRa. The RNA samples were subjected to RNAseq analyses. (a) The differentially expressed genes between any two experimental groups (FC ≥ 4, Padj < 0.05) were selected for heatmap analyses. Genes with overall distinctive expression patterns were highlighted

by colored boxes. (b) The expression patterns for a number of classical p53 targets were extracted from the dataset. The corresponding FPKM values are shown in a bar graph (mean±SEM, n=3 biological replicates). (c) Via comparisons between the sgNC and sgIfn groups (of the parental LLC cells), the genes up-regulated in sgIfn group were selected (FC ≥ 4, Padj < 0.05). The gene list was subjected to GO analyses. **(d)** Co-cultures containing LLC cells (labeled with mCherry) and their p53^{+} knock-in derivatives were established. The P_{Suv}-CRISPRa-Ifng/P_{M2}-ACRmax circuit (a circuit with sgNC as a control) were packaged using a group of two lentiviral vectors (non-fluorescent, LV_{NF}). The co-cultured cells were transduced with the LV_{NF} (left illustration). At day-7 after transduction, cells were subjected to fluorescent sorting, where the mCherry^{+} LLC cells and mCherry^{-} p53^{+} cells were separated (middle flow cytometry panel). The RNA samples from the sorted cells were analyzed by qPCR [mean±SEM, n=3 biological replicates] (right). **(e-g)** (e) As illustrated on the left, the P_{Suv}-CRISPRa-*Ifng*/P_{M2}-ACRmax circuit (a circuit with sgNC as a control) were packaged using another group of two lentiviral vectors with fluorescent labels (LV_{FL}, with either an mCherry or EGFP label). They were used to transduce either the LLC cells or their p53-derivatives *in vitro*. Seven days after transduction, the cells were analyzed on flow cytometry for circuit introduction efficiency (the mCherry^{+}EGFP^{+} cells). (f) The RNA samples from transduced cells were analyzed by qPCR. The levels from 3 independent experiments are presented, with the black lines denoting the mean values. (g) The *in vitro* growth profile by these cells were determined by MTT (eight biological replicates, mean±SD). **(h)** With the *in vitro* characterizations, the circuit

introduced cells (no selection, 2x10^6) were further implanted subcutaneously to the flanks of mice. At day-7 and day-17 after tumor cell inoculation, some tumors were harvested for flow cytometry analyses for the numbers of $CD8^+$ T cells (in the $CD45^+$ population). Mean values (±SEM) are shown (For day-7, “sgNC”: n=4, the other three groups: n=3; for day-17, “sgNC”: n=5, the other three groups: n=6 tumor samples). Source data are provided in the Source Data file.

Supplementary Table 1: Reagents or resources

Reagents	Source	Identifier
**Antibodies [dilution in use]**		
anti-spCas9 [1:500]	GenScript	A01935-40
anti-GFP [1:2000]	Abcolonal	AE012
anti-STAT1 [1:1000]	Sangon Biotech	D155186
anti-p-STAT1 (Y701) [1:1000]	Cell Signaling Technology (CST)	7649S
anti-GAPDH [1:1000]	Santa Cruz	SC32233
anti-Actin [1:1000]	GenScript	A00730
anti-Flag [1:1000]	Sigma	F1804
anti-p53 [1:500]	Santa Cruz	SC126
APC anti-human CD45 [1:100]	BioLegend	304012
FITC anti-mouse/human CD11b [1:100]	BioLegend	101205
FITC anti-human HLA-A, B, C [1:100]	BioLegend	311404
PE anti-human HLA-DR [1:100]	BioLegend	307606
APC/Cyanine7 anti-mouse CD45 [1:100]	BioLegend	103116
FITC anti-mouse CD4 [1:100]	BioLegend	100406
BV421 anti-mouse CD8a [1:100]	BioLegend	100737
APC anti-mouse CD3ε [1:100]	BioLegend	100311
LIVE/DEAD Cell Stain Kit [1:1000]	Invitrogen	L34965
**Cytokines, Chemicals**		
recombinant human IFNα2	Peprotech	300-02AA-100
recombinant human IFNγ	Peprotech	300-02
recombinant mouse TNFα	Sangon Biotech	C600052
recombinant human M-CSF	PeproTech	300–25
Cobalt(II) chloride hexahydrate ($CoCl_2$)	Sigma	C8611
Doxycycline	Sigma	D9891
Dual-luciferase	Pierce	E1910
Puromycine	Sangon Biotech	PJ593
Blasticidin	InvivoGene	ant-bl
Thiazolyl Blue Tetrazolium Bromide	Yeasen	40201ES72
Histopaque-1077	GE Healthcare	17-5442-02
Polybrene	Sigma	TR-1003-G
Nutlin-3	Sigma	N6287
Cisplatin	Sigma	PHR1624
Lipofectamine 3000 Transfection kit	Invitrogen	L3000-015
**Cell Culture Reagents**		
DMEM	Gibco	12100-046
RPMI-1640	Gibco	31800-022
Fetal bovine serum	Gibco	10099-141
Pen/Strep	Gibco	15140-122
Opti-MEM	Gibco	31985-070
0.25% Trypsin-EDTA	Gibco	25200-072
HEPES	cytiva	SH30237.01
**Cell lines**		
HEK 293T	ATCC	CRL-3216
NCI-H1299	ATCC	CRL-5803
A549	ATCC	CCL-185
Lewis lung carcinoma (LLC)	ATCC	CRL-1642

Supplementary Table 2: Spacer sequence of gRNAs

Name	sequence
sgCUT	GCTTGTAGATGGCCATGGCG
sgCon-i	TAGGCGTTTTGCGCTGCTTC
sgCon-a (sgBsaI)	TGAGACCGAGAGAGGGTCTCA
sgTGTi and sgTGTa	GTCAGATCCGCTAGCGCTAC
sgRenilla	GATGATAACTGGTCCGCAG
sg#1-i	AACTAGGGAAAGTGAAACTA
sg#2-i	GAACACAAAGCATAGACTGC
sg#3-i	GGCCCTGCAATGTCAAGGGA
sg#5-i	ACACGCCTAGAGTCTCCGCA
sg#4-i	GTGCTAGCTCGGCGATCTGA
sgNC	Used as a negative control for endogenous genes - same as "sgTGTa"
sghIFNG-1	TCTCATCGTCAAAGGACCCA
sghIFNG-2	GAATCCCACCAGAATGGCAC
sghIFNG-3	GAGATGGTGACAGATAGGCA
sghIFNG-4	GAAGAGTCAACATTTTACCA
sghCCL21-1	CTTTCAGGAAGGAAAAGGAG
sghCCL21-2	TGGGAATAGAAGGAAGGCTC
sghCCL21-3	GCGTAGTGAGGAGACAGTCA
sgmIfng-5	GTTACTTTGCATTACAGCTA
sgmIfng-8	ATCCCGAGGAGCCTTCGATC
sgmp53 for KI	GTATGGCGGGATGTATCTTA
sgTemplate	GAGATCGAGTGCCGCATCAC

Supplementary Table 3: Genotyping primers

Primer Name	Sequence	Size	Note
0036	ACAGCGTGGTGGTACCTTAT	KO 650bp	*p53* $^{+/-}$ mice
0037	TATACTCAGAGCCGGCCT	WT 450bp	*p53* $^{+/-}$ mice
0038	CTATCAGGACATAGCGTTGG		*p53* $^{+/-}$ mice
Ptempl-F	CGTCGCGATCGGAAATGAGA		*p53* $^{+}$ LLC cell line
Pgeno-R	AAAGGAATGTGAGGGAAGAGAGTTC	1262bp	*p53* $^{+}$ LLC cell line

Supplementary Table 4: qPCR primers

Gene Name	Forward primer （5` to 3`）	Reverse primer （5` to 3`）
human CDKN1A	AGGTGGACCTGGAGACTCTCAG	TCCTCTTGGAGAAGATCAGCCG
human MDM2	TGTTTGGCGTGCCAAGCTTCTC	CACAGATGTACCTGAGTCCGATG
human IFNG	TCGGTAACTGACTTGAATGTCCA	TCGCTTCCCTGTTTTAGCTGC
human CCL21	TACCGGAAGCAGGAACCAAG	GTGGGGATGGTGTCTTGTCC
human GAPDH	GGAGCGAGATCCCTCCAAAAT	GGCTGTTGTCATACTTCTCATGG
mouse Ifng	CAGCAACAGCAAGGCGAAAAAGG	TTTCCGCTTCCTGAGGCTGGAT
mouse Irf1	TCCAAGTCCAGCCGAGACACTA	ACTGCTGTGGTCATCAGGTAGG
mouse Cdkn1a	TCGCTGTCTTGCACTCTGGTGT	CCAATCTGCGCTTGGAGTGATAG
mouse Actb	TGCTGGAAGGTGGACAGTGAGG	TGCTGGAAGGTGGACAGTGAGG
mouse Cxcl10	ATCATCCCTGCGAGCCTATCCT	GACCTTTTTTGGCTAAACGCTTTC
mouse CD8a	ACTACCAAGCCAGTGCTGCGAA	ATCACAGGCGAAGTCCAATCCG
mouse IL2ra	GCGTTGCTTAGGAAACTCCTGG	GCATAGACTGTGTTGGCTTCTGC
mouse Prf1	AGCACAAGTTCGTGCCAGG	GCGTCTCTCATTAGGGAGTTTTT
mouse Gzmb	CCACTCTCGACCCTACATGG	GGCCCCCAAAGTGACATTTATT
mouse Cd69	GGGCTGTGTTAATAGTGGTCCTC	CTTGCAGGTAGCAACATGGTGG
mouse Stat1	GCCTCTCATTGTCACCGAAGAAC	TGGCTGACGTTGGAGATCACCA
mouse H2-D1	GCTGCAGAGCATTACAAGGC	GCCAGGTCAGGGTGATGTC
mouse H2-Ab1	AGCCCCATCACTGTGGAGT	GATGCCGCTCAACATCTTGC
mouse Pdcd1lg1	TGCGGACTACAAGCGAATCACG	CTCAGCTTCTGGATAACCCTCG
mouse Gapdh	CATCACTGCCACCCAGAAGACTG	ATGCCAGTGAGCTTCCCGTTCAG
