# Supplementary material for: Precise tumor immune rewiring via synthetic CRISPRa circuits gated by concurrent gain/loss of transcription factors
Source: Nat Commun. 2022 Mar 18;13:1454. doi: 10.1038/s41467-022-29120-y (PMC8933567; doi:10.1038/s41467-022-29120-y)
Supplement: Supplementary file 2 — Reporting Summary [file 41467_2022_29120_MOESM2_ESM.pdf]

nature portfolio

Corresponding author(s): Jianghuai Liu, Xingxu Huang, Geng Liu

Last updated by author(s): Feb 24, 2022

Reporting Summary

Nature Portfolio wishes to improve the reproducibility of the work that we publish. This form provides structure for consistency and transparency in reporting. For further information on Nature Portfolio policies, see our Editorial Policies and the Editorial Policy Checklist.

Statistics

For all statistical analyses, confirm that the following items are present in the figure legend, table legend, main text, or Methods section.

n/a	Confirmed	
☐	☒	The exact sample size (*n*) for each experimental group/condition, given as a discrete number and unit of measurement
☐	☒	A statement on whether measurements were taken from distinct samples or whether the same sample was measured repeatedly
☐	☒	The statistical test(s) used AND whether they are one- or two-sided<br>*Only common tests should be described solely by name; describe more complex techniques in the Methods section.*
☒	☐	A description of all covariates tested
☒	☐	A description of any assumptions or corrections, such as tests of normality and adjustment for multiple comparisons
☐	☒	A full description of the statistical parameters including central tendency (e.g. means) or other basic estimates (e.g. regression coefficient) AND variation (e.g. standard deviation) or associated estimates of uncertainty (e.g. confidence intervals)
☐	☒	For null hypothesis testing, the test statistic (e.g. *F*, *t*, *r*) with confidence intervals, effect sizes, degrees of freedom and *P* value noted<br>*Give P values as exact values whenever suitable.*
☒	☐	For Bayesian analysis, information on the choice of priors and Markov chain Monte Carlo settings
☒	☐	For hierarchical and complex designs, identification of the appropriate level for tests and full reporting of outcomes
☒	☐	Estimates of effect sizes (e.g. Cohen's *d*, Pearson's *r*), indicating how they were calculated

Our web collection on statistics for biologists contains articles on many of the points above.

Software and code

Policy information about availability of computer code

Data collection	Illumina HiSeq platform, FACSDiva (8.0.1), CellQuest Pro (5.2.1)
Data analysis	FlowJo (X 10.07r2), CasOFFinder (2.4), CRISPR-ERA, GenSmart Codon Optimizer, DESeq2 (Bc 3.14), PANTHER (17.0), ImageJ (1.53i)

For manuscripts utilizing custom algorithms or software that are central to the research but not yet described in published literature, software must be made available to editors and reviewers. We strongly encourage code deposition in a community repository (e.g. GitHub). See the Nature Portfolio guidelines for submitting code & software for further information.

Data

Policy information about availability of data

All manuscripts must include a data availability statement. This statement should provide the following information, where applicable:

- Accession codes, unique identifiers, or web links for publicly available datasets
- A description of any restrictions on data availability
- For clinical datasets or third party data, please ensure that the statement adheres to our policy

High-throughput RNAseq data (raw and processed) are deposited at the NCBI GEO database with the accession number of GSE179814: (https://ncbi.nlm.nih.gov/geo/query/acc.cgi?acc=GSE179814). The reference mouse genome assembly Mus_musculus.GRCm38 is an openly accessible resource (https://www.ncbi.nlm.nih.gov/assembly/GCF_000001635.20/). Source data for the presented results are provided with this paper.

Field-specific reporting

Please select the one below that is the best fit for your research. If you are not sure, read the appropriate sections before making your selection.

- [x] Life sciences
- [ ] Behavioural & social sciences
- [ ] Ecological, evolutionary & environmental sciences

For a reference copy of the document with all sections, see nature.com/documents/nr-reporting-summary-flat.pdf

Life sciences study design

All studies must disclose on these points even when the disclosure is negative.

Sample size	Samples sizes were indicated in figure legends. No statistical methods were used to predetermine sample sizes. The sample sizes for our experiments were based on related studies in the field (see Refs: Nissim L, Wu M, Pery E et al., 2017 [10.1016/j.cell.2017.09.049]; Nakamura M, Srinivasan P, Chavez M et al., 2019 [0.1038/s41467-018-08158-x]; Wang G, Chow RD, Bai Z et al., 2019 [10.1038/s41590-019-0500-4]; Chung HK, Zou X, Bajar BT et al., 2019 [10.1126/science.aat6982]).
Data exclusions	No data has been excluded from the analyses.
Replication	As indicated in the figure legends, all data represented have been validated in biological replicates and through independent experiments (at least two). The average values from such replications are presented (+/- STD or SEM or range). The reported results are highly reproducible.
Randomization	C57/BL6 mice of the same age and sex were randomly divided into cohorts for parallel inoculation with different groups of tumor cells. They were further housed under the same conditions. Randomization was not applied to in vitro cell line experiments, as minimal covariates are expected to be associated with cell assays.
Blinding	Blinding was not applied. For most of the experiments, a series of samples representing different groups are analyzed side-by-side in a single trial. All measurements in this study are objective (fluorescent readings, differential band intensities, tumor sizes etc). In most cases, differences between given groups are demonstrated in relation to internal controls (e.g., loading or transfection controls). Correlations in different types of measurements provide further confidence to the conclusions made. Moreover, experimental replications are performed to exclude artifacts.

Reporting for specific materials, systems and methods

We require information from authors about some types of materials, experimental systems and methods used in many studies. Here, indicate whether each material, system or method listed is relevant to your study. If you are not sure if a list item applies to your research, read the appropriate section before selecting a response.

Materials & experimental systems

n/a	Involved in the study	
☐	☒	Antibodies
☐	☒	Eukaryotic cell lines
☒	☐	Palaeontology and archaeology
☐	☒	Animals and other organisms
☒	☐	Human research participants
☒	☐	Clinical data
☒	☐	Dual use research of concern

Methods

n/a	Involved in the study	
☒	☐	ChIP-seq
☐	☒	Flow cytometry
☒	☐	MRI-based neuroimaging

Antibodies

Antibodies used

For Western blots:
spCas9 (GenScript, A01935-40);
GFP (Abclonal, 9200012003);
STAT1 (Sangon Biotech, AB55186);
p-STAT1 (Y701, CST 7649S);
GAPDH (Santa Cruz SC32233);
Actin (GenScript, A00730);
Flag (Sigma F1804);
and p53 (Santa Cruz SC126); and see Methods and Supplementary Table 1 for dilutions.

Antibodies for flow cytometry from Biolegend :
APC anti-human CD45 (304012);
FITC anti-mouse/human CD11b (101205);
FITC anti-human HLA-A/B/C (311404);

	PE anti-human HLA-DR (307606);<br>APC/Cyanine7 anti-mouse CD45 (103116);<br>FITC anti-mouse CD4 (100406);<br>BV421 anti-mouse CD8a (100737);<br>APC anti-mouse CD3ε (100311).
Validation	All antibodies used in this study are validated by the manufacture (see their respective website for validation and citations).<br>Briefly, the "spCas9" antibody is validated for WB and IF;<br>the GFP antibody is validated for WB, IF;<br>the Stat1 antibody is validated for WB, IHC;<br>the pStat1 antibody is validated for WB, IP, IF, ChIP, FC;<br>the GAPDH antibody is validated for WB, IF, IP;<br>the Actin antibody is validated for WB, ELISA;<br>the Flag antibody is validated for WB, ELISA;<br>and the p53 antibody is validated for WB, IP, IF, FC.<br><br>The endogenous pathways associated with this study are all well characterized (IFN and p53 pathways). Some antibodies (STAT1, phosphor-STAT1, GAPDH and Actin) have been validated in our previous study (Tong Y. et al., EBioMedicine 2019). The p53 antibody is validated by the inducible transgene expression and by the knockout MEF cells (Fig. 3 and 4). Other antibodies are associated with transfected genes (Cas9, EGFP, Flag, HA). Their specificity were validated by non-transfected or untreated controls in Western blots.<br><br>In flow cytometry, antibodies for CD45, CD11b, HLA-ABC, HLA-DR, CD3, CD4 and CD8 had been validated for FC analyses by the manufactures. Citations for the antibodies can be found on the products' website (Biolegend).

Eukaryotic cell lines

Policy information about cell lines

Cell line source(s)	293T (CRL-3216), H1299 (CRL-5803), A549 (CCL-185), LLC (CRL-1642) cells were obtained from ATCC.
Authentication	293T cells were not authenticated in this study. Other recently obtained cell line (H1299, A549, LLC) were authenticated by the supplier by STR typing. The H1299 cells were further validated during the course of this study by the absence of p53 protein and activities (Fig. 3 and Fig. S4D). A549 was further validated in the lab by its mutation at Kras gene (causing G12S, not shown). LLC was further validated during the cause of the study by an established mutation at p53 (causing R334P).
Mycoplasma contamination	All cell lines were tested negative of mycoplasma contaminations.
Commonly misidentified lines<br>(See ICLAC register)	None of the cell lines used are listed in the ICLAC database.

Animals and other organisms

Policy information about studies involving animals; ARRIVE guidelines recommended for reporting animal research

Laboratory animals	All mice used were of C57/BL6 background. The mice were housed in a humidity- and temperature-controlled, specific pathogen-free facility under a 12:12 h light/dark cycle. The p53+/− mice were originally obtained from the Jackson lab. The male and female mice were crossed for generation of p53-/- embryos. Primary mouse embryonic fibroblasts (MEF) of different genotypes were obtained from 13.5-14.5 d embryos. C57BL/6J mice at 6-8 weeks of age were used for the in vivo transplantable tumor model.
Wild animals	This study does not involve wild animals.
Field-collected samples	This study does not involve field-collected samples.
Ethics oversight	The animal experiments were approved by the Institutional Animal Care and Use Committee of Model Animal Research Center of Nanjing University (MARC-NJU).

Note that full information on the approval of the study protocol must also be provided in the manuscript.

Flow Cytometry

Plots

Confirm that:

- [x] The axis labels state the marker and fluorochrome used (e.g. CD4-FITC).
- [x] The axis scales are clearly visible. Include numbers along axes only for bottom left plot of group (a 'group' is an analysis of identical markers).
- [x] All plots are contour plots with outliers or pseudocolor plots.
- [x] A numerical value for number of cells or percentage (with statistics) is provided.

Methodology

Sample preparation	The adherent cells (cell line cultures) were either trypsinized (with GFP reporter) or non-enzymatically dissociated (PBS containing 0.5% EDTA). For measuring cell-surface antigen levels, the cell suspensions were subsequently stained with fluorochrome-labeled antibodies for 1 hour on ice. After washing, the cells were subjected to flow cytometry analyses. To analyze the immune compartment in the tumors, the samples were prepared as describe before. Briefly, the tumor was cut into small pieces and was further dissociated with the help of enzymatic digestion (collagenase I [170mg/L], collagenase II [56mg/L] and DNase I [25mg/L]) for 30 min at 37°C. The filtered (via 80 µm nylon mesh) cell suspension was next subjected to red blood cell removal using the ACK buffer. The cells were washed with PBS twice before proceeded to antibody staining.
Instrument	BD LSRFortessa or BD FACS Calibur
Software	FACSDiva (8.0.1), CellQuest Pro (5.2.1) for collections, and FlowJo (X 10.07r2) for analyses
Cell population abundance	For analyses of Class II HLA levels in PBMC, sufficient total cells that contained around 10,000 in the CD45+CD11b+ compartment were collected. For analyses of Class I HLA levels in cultured tumor cells, a total of around 30,000 cells were analyzed. For analyses of EGFP reporter, a total of around 25,000 (H1299, 293T), 60,000 (A549) or 200,000 (LLC) cells were analyzed. For analyses of tumor immune compartment in each sample, around 100,000 of CD45+ cells were analyzed. CD8+ T cells comprise around 2% within the CD45+ population.
Gating strategy	In cells transfected with the activatable EGFP reporter, the predominant cell populations in the FSC/SSC plot were first selected. The untransfected cells were used as references to confirm the clear separation of EGFP- (mininal basal fluorescence) and EGFP+ cells. The fluorescent patterns of EGFP+ cells are presented. To determine cell surface Class I HLA levels in cultured tumor cells, the predominant cell populations in the FSC/SSC plot were first gated. For PBMC, cells debris were first excluded from analyses using the FCS/SSC plot. The remaining cells were gated by CD45 and CD11b. The HLA-DR levels in the CD45+CD11b+ monocyte compartment are presented. In analyses of tumor CD8 T cells, the single cells from the tumors were gated through Aqua405-CD45+CD3+CD8+. This gating strategy is shown in the Supplementary Fig. 6.

☒ Tick this box to confirm that a figure exemplifying the gating strategy is provided in the Supplementary Information.
