# Supplementary material for: Precise tumor immune rewiring via synthetic CRISPRa circuits gated by concurrent gain/loss of transcription factors
Source: Nat Commun. 2022 Mar 18;13:1454. doi: 10.1038/s41467-022-29120-y (PMC8933567; doi:10.1038/s41467-022-29120-y)
Supplement: Supplementary file 4 — Description of Additional Supplementary Files [file 41467_2022_29120_MOESM4_ESM.pdf]

Title: Supplementary Data 1:
Description: Sequences from key constructs.
